# 340. Outcomes of COVID-19 in Hospitalized SOT Recipients: Experence in Colombia, South America

**DOI:** 10.1093/ofid/ofab466.541

**Published:** 2021-12-04

**Authors:** Fernando Rosso, Eric Tafurt

**Affiliations:** 1 Fundación Valle del Lili Universidad Icesi, Cali, Valle del Cauca, Colombia; 2 Fundacion Valle del lili, Cali, Valle del Cauca, Colombia

## Abstract

**Background:**

SOTs (SOT) recipients with COVID‐19 are considered to be at high risk of severe clinical outcomes. Several descriptive studies have reported a high frequency of intensive care unit admission and death rates. There is a lack of evidence regarding the best approach for immunosuppressive therapy in SOT recipients with COVID-19.

**Methods:**

We performed a single-centered, retrospective, observational study of all SOT recipients with SARS-CoV-2 confirmed infection RT-PCR from nasopharyngeal swab specimens who were admitted to the emergency department from March 25 to September 1, 2020. Glucocorticoid therapy was administered according to the criteria of the attending physician. We classified glucocorticoid dose as low dose therapy if the patient received dexamethasone 6 mg/day or methylprednisolone 40 mg/day, and a high dose if the patient received methylprednisolone 80–160 mg/day. Specimens collected within the first 48 hours

were defined coinfection, while specimens collected after 48 hours were defined as hospital-acquired superinfection.

**Results:**

Of a total of 43 SOT recipients with COVID-19, 17 (39%) required intensive care unit admission. 32 (74.4%) required glucocorticoid therapy: 13 received low dose and 19 high dose. 15 (34.8%) had secondary infections. A total of 12 (27.9%) presented hospital-acquired bacterial superinfections, mostly caused by P. aeruginosa, most of isolations were from respiratory tract cultures. The median time from hospital admission to superinfection diagnosis was 9 (7-13) days. Community-acquired co-infection at COVID-19 diagnosis was documented only in 3 (6.9%) patients, mostly caused by K. Pneumoniae, all isolations were from urine culture. Glucocorticoid therapy was indicated in 32 (80%) patients, 19 received high dose and 13 low doses. Overall hospital mortality was 17.5%. ICU mortality was 41%. Overall mortality in the high dose steroids group was 37 % vs . 0% in the low dose group.

**Conclusion:**

Our results showed a higher frequency of superinfection in SOT recipients with COVID-19 compared to previous reports, and higher ICU mortality. Further studies are needed to establish the best approach for glucocorticoid therapy in SOT recipients with COVID-19.

**Disclosures:**

**All Authors**: No reported disclosures

